# Profundae diversitas: the uncharted genetic diversity in a newly studied group of fungal root endophytes

**DOI:** 10.1080/21501203.2015.1070213

**Published:** 2015-07-24

**Authors:** Brian R. Murphy, Lucia Martin Nieto, Fiona M. Doohan, Trevor R. Hodkinson

**Affiliations:** a School of Natural Sciences & Trinity Centre for Biodiversity Research, Trinity College Dublin, College Green, Dublin 2, Ireland; b Agricultural and Environmental Sciences Faculty, Salamanca University, 37007Salamanca, Spain; c UCD Earth Institute and School of Biology & Environmental Science, University College Dublin, Dublin 4, Ireland

**Keywords:** *Hordeum murinum*, fungal root endophytes, genetic diversity, biofertilisation, biocontrol

## Abstract

Endophytes associated with crops have potential as beneficial inoculants in agriculture, but little is known about their genetic diversity and phylogenetic relationships. We carried out the first ever ecological and phylogenetic survey of the culturable fungal root endophytes of a wild barley species. Fungal root endophytes were isolated from 10 populations of wall barley (*Hordeum murinum*), and 112 taxa of fungi were identified based on internal transcribed spacer sequence similarity. We found representatives from 8 orders, 12 families and 18 genera. Within this group, only 34 isolates (30% of the total) could be confidently assigned to a species, and 23 of the isolates (21% of the total) had no significant match to anything deposited in GenBank (based on <85% sequence similarity). These results suggest a high proportion of novel fungi, with 28% not assigned to a known fungal order. This includes three endophytes that have been shown to significantly improve agronomic traits in cultivated barley. This study has, therefore, revealed a profound diversity of fungal root endophytes in a single wild relative of barley. Extrapolating from this, the study highlights the largely unknown, hugely diverse and potentially useful resource of crop wild relative endophytes.

## Introduction

Endophytic fungi are phylogenetically diverse and occur nearly ubiquitously in land plants across a broad range of ecosystems (Weiss et al. 2004, 2011; Riess et al. 2014). Definitions of what constitutes an endophyte vary (Schulz and Boyle 2006; Wang et al. 2009; Murphy 2013), but they can be broadly defined as micro-organisms that can live at least part of their life cycle inter- or intracellularly inside plants, usually without inducing pathogenic symptoms. This can include competent, facultative, obligate, opportunistic and passenger endophytes. Endophytes can have several functions and/or may change function during their life cycle (Murphy et al. 2013, 2014a). Benefits to crop plants infected with endophytic fungi include an increase in seed yield (Achatz et al. 2010; Murphy et al. 2014b, 2015), enhanced resistance to pathogens and herbivores (Cheplick and Faeth 2009; Murphy et al. 2015a) and increased stress tolerance (Waller et al. 2005; Rodriguez et al. 2009).

In recent years, DNA-based studies of endophytic organisms isolated from the wild and cultivated plant systems have led to an increasing level of awareness of the high phylogenetic diversity within fungal endophytes (Andrade-Linares et al. 2011; Sánchez Márquez et al. 2012; Ek-Ramos et al. 2013). Some of these endophytes are localised to specific tissue types and some are found systemically. The leaf endophytes of many grassy plant species, including barley (*Hordeum vulgare*) have been well studied and characterised (Arnold and Lutzoni 2007; Cheplick and Faeth 2009; Wang et al. 2009; Torres et al. 2012; de Souza Leite et al. 2013). The endophytes of wild barley species have been less studied, particularly root endophytes. Endophyte-induced resistance to aphid shoot infestation has been demonstrated in *H. brevisubulatum* subsp. *violaceum* and *H. bogdanii* (Clement et al. 1997) and *Neotyphodium* endophytes have been shown to readily colonise the leaves of *H. brevisubulatum* subsp. *violaceum* (Dugan and Sullivan 2002). Investigations focusing on the communities inhabiting roots are extremely limited and, to our knowledge, no previous studies have examined the genetic diversity of fungal root endophytes isolated from a wild relative of a major cereal crop.

*Hordeum murinum* (wall barley) is a wild species native to Central Europe, the Mediterranean region, North Africa, south-western Asia, the Caucasus, southern Uzbekistan, Tadzjikistan, Iran and Afghanistan (Von Bothmer et al. 1995). Genetic studies permit the recognition of three subspecies: subsp. *glaucum*, subsp. *leporinum* and subsp. *murinum* (Booth and Richards 1976, 1978; Richards and Booth 1977; Giles and Lefkovitch 1986). It has been introduced as a weed to most parts of the world, especially subsp. *glaucum* and subsp. *leporinum*. The original habitats were probably seasides, sandy riverbeds and rough disturbed ground. Presently, it is common as a weed in all places with human disturbance. The tetraploid *H. murinum* subsp. *murinum* (2*n* = 28) is the subspecies encountered most often in northern and western Europe north of the Mediterranean (Streeter et al. 2009; Jakob and Blattner 2010). In Ireland, *H. murinum* is an archaeophyte and is a ruderal of roadsides, rough grassland and waste places, almost certainly introduced by man (Cope and Gray 2009). It is chiefly found near towns in the drier eastern and southern half of the island; it is common around Dublin but rare elsewhere (Scannell and Synnott 1987; Poland and Clement 2009; Streeter et al. 2009; Stace 2010; Parnell and Curtis 2012). The population genetics of *H. murinum* in Ireland has not been studied in any detail and we do not know how Irish populations differ genetically from other European populations.

Murphy et al. (2015a, 2015b) have shown that some of the endophytes isolated from *H. murinum* can have significant agronomic benefits for cultivated barley grown under a variety of biotic and abiotic stresses, so the untapped potential for other endophytes within this group may be great. We know almost nothing about the diversity of endophyte communities within *H. murinum*. In the current study, we carried out the first comprehensive genetic survey of the culturable fungal root endophytes (hereafter endophytes) of *H. murinum*. We examined the nuclear ribosomal internal transcribed spacer (ITS) DNA variation (nrITS) of isolated fungal strains from 10 *H. murinum* populations in Ireland and assessed their identity at various taxonomic ranks using similarity searches in the National Center for Biotechnology Information (NCBI) Basic Local Alignment Search Tool (BLAST). We also examined some ecological variables of the sample sites to estimate if endophyte variability was related to ecological variables.

## Materials and methods

### Site and plant selection

Whole plants of *H. murinum* were collected from 10 urban and suburban populations from within a 10 km radius of a point centred in Ireland at 53.39602N, 6.21632W in June–July. Root samples were collected from a minimum of 10 plants per population. Environmental and plant variables were recorded at the time of collection and included: soil pH, soil salinity (measured as osmotic potential in bars), soil moisture content, plant height (cm), Zadoks growth stage (Zadoks et al. 1974) and plant health (scored on a five point scale, with a score of five indicating large plants of excellent health with no apparent disease or physiological stress symptoms and a score of zero indicating plants with severe disease or stress symptoms). The overall vegetation type and soil type for each site were assessed using a numerical equivalent. For vegetation type: 1 = a site without any significant soil and with no other vegetation (for example the edge of a roadside kerb), 2 = an open site with short grass, 3 = an open site with short weedy vegetation, 4 = a site with shading from deciduous trees and short grass, 5 = a site at the base of a wall with no other vegetation. For soil type: 1 = a light sandy silt, 2 = a light sandy loam, 3 = a dark clay loam with few stones. Data analysis of ecological variables was carried out using single and two-factor analysis of variance (ANOVA) with Bonferroni correction and Pearson’s correlation statistical analyses supplied with the Data Analysis module within Microsoft Excel® and Datadesk 6.1®.

### Endophyte isolation

Roots were separated from whole plants and surface-sterilised in 5% NaClO for 15 min then rinsed five times with sterile water. Ten root pieces of 5 mm length from each plant were inoculated onto culture plates of malt extract agar (Sigma-Aldrich Fluka 38954 modified MEA, Vegitone) and incubated in the dark at 25°C for 28 days. This medium is recommended by Sigma-Aldrich for the isolation, detection and enumeration of yeasts and moulds. The animal derived peptone of the original formulation is replaced by a plant peptone. The powdered medium was mixed to half-strength of the manufacturers’ recommendations (to avoid osmotic shock to the endophytes) using pure water then sterilised by autoclaving. From previous experience, we considered 28 days to be sufficient time to allow recovery of the slowest emerging endophytes. Dishes were inspected daily and those containing root pieces with surface fungal growth were discarded (i.e. not emerging from the cut root area). Emergent endophytes were removed and subcultured on the same medium in the dark at 25°C for further 14 days.

### DNA extraction and ITS sequencing

For the DNA analysis, 20 mg of fungal material was scraped from the agar surface and placed into shaker tubes. DNA was extracted using a Qiagen DNeasy mini kit, following the Qiagen protocol, producing 200 µl of DNA extract for each isolate. Polymerase chain reaction (PCR) was carried out on the DNA extracts using the nrITS primers ITS1 and ITS4 (White et al. 1990). The PCR reaction contained 1 μl of DNA extract (ca. 20 ng μl^−^^1^), 5 μl of 5× buffer (Promega), 0.5 μl of 10 mM dNTPs, 0.25 μl of 20 pmol μl^−^^1^ forward primer), 0.25 μl of each primer at 20 pmol μl^−^^1^, 2 μl of 25 mM MgCl_2_ and 0.125 μl of Go Taq Flexi DNA polymerase (Promega). Thermal cycling in an Applied Biosystems Veriti® thermal cycler included a premelt of 94°C for 30 sec followed by 32 cycles of 94°C for 2 min, 57°C for 1 min and 72°C for 1 min, followed by a final extension of 7 min at 72°C. PCR products were purified using Exonuclease (New England Biolabs) and Shrimp Alkaline Phosphatase (ExoSAP; Roche). Purified PCR products underwent cycle sequencing using the reverse ITS4 primer or forward ITS1 primer in separate reactions using the Applied Biosystems BigDye 3.1 kit (Foster City, CA) and the manufacturer’s instructions. The products were further purified using an Applied Biosystems BigDye XTerminator purification kit and protocol (Foster City, CA). DNA was sequenced using an Applied Biosystems 3130xL Genetic Analyzer.

### Sequence analysis

A total of 112 isolate sequences were recovered and compared with GenBank accessions using the megaBLAST, and identified using morphological and DNA characters. BLAST similarity criteria for assigning taxonomic rank to the endophyte strains was allocated based on an initial survey of existing fungal taxa in GenBank, as follows: >97% similarity was assigned to the same species, 90–96% to the same genus, 85–90% to the same order and <85% to no significant match. In all cases, genetic identity assignment was confirmed or further assessed by examination of morphological characters of the fungi using light microscopy and by referencing the taxonomic descriptions found in Cannon and Kirk (2007).

Every recovered sequence from our sampling was combined into a matrix and analysed to detect recombination events using the recombination analysis tool (RAT) (Etherington et al. 2005), which uses the distance-based method of recombination detection. Window size was set to 93 characters (0.1 of sequence length) and Increment size was 46 characters (0.5 of Window size). For each fungal order represented by our recovered endophytes, we selected reliable GenBank accessions based on identification to species level, relatively large number of characters, reputation of originator and publication status. The recovered sequences were grouped into fungal orders and combined with the selected GenBank accessions into a final sequence matrix for each order. Analysis at order level was conducted because it was not possible to reliably align sequences in matrices above this taxonomic rank. The sequence matrices were aligned using the MUSCLE alignment algorithm (Edgar 2004) on the Molecular Evolutionary Genetics Analysis (MEGA) 6.0 platform (Tamura et al. 2013) with the following parameters: gap open penalty = −400, gap extend penalty = 0, max iterations = 8, clustering method = Unweighted Pair Group Method with Arithmetic Mean and max diag length (lambda) = 24. The matrices were further refined by examination and manual realignment, and suspected stuttering sections (microsatellite repeat regions) in sequences were removed. A phylogeny was reconstructed for each order using MEGA 6.0 to construct a maximum likelihood (ML) phylogenetic tree, with a bootstrap test of phylogeny (2500 replications). The Kimura 2-parameter model was selected with uniform rates between sites, partial deletion of gaps/missing data and a 95% site coverage cut-off. Nearest-Neighbour_Interchange (NNI) was selected as the ML heuristic method, with a very strong branch swap filter and an Neighbour Joining (NJ)/BioNJ initial tree.

The overall mean distance (the mean pairwise distance and standard error for the set of sequences) and diversity for each fungal order alignment, calculated as the number of base substitutions per site, was estimated using the ‘Distance’ and ‘Diversity’ functions in MEGA 6.0 with the following parameters: maximum composite likelihood model, including d:transitions + transversion substitutions, uniform rates among sites, a homogeneous pattern among lineages and pairwise deletion of gaps/missing data. The sequences were then split into two groups, (1) the sequences recovered from our sampling and (2) the selected GenBank accessions, and mean distance and diversity within and between these groups was calculated using the same parameters. An overall coefficient of differentiation (the estimate of the proportion of interpopulational diversity) for the combined groups was also calculated.

The final phylogenetic trees were annotated using Figtree v1.4.0 (Rambaut 2012), and formatted using Microsoft PowerPoint®.

## Results

### Ecological variation in host establishment and endophyte abundance

All of the sampling sites were characterised by a relatively high soil salinity (mean = 1.37 bars), high soil pH (mean 7.7) and low soil moisture content (mean 10.7%), with four sites having no measurable soil moisture (Table 1). Seven sites had the same soil type: a light sandy silt, with relatively low soil moisture content (13% or less). The host plants were from 20 to 63 cm in height, erect or spreading and occurred as occasional annual roadside and waste ground ruderals in towns and suburbs. The populations that we studied show that *H. murinum* prefers light, dry and basic/alkaline soils with little competition. All of the plants were harvested at a similar stage and had reached at least the early flowering stage (Zadoks growth stage 59), but no plants had completed anthesis. There was no significant correlation between Zadoks growth stage and any other parameter. In total, 164 individual endophyte isolates were recovered, with three sites (location 5, 7 and 9) accounting for half of these. We found a significant variation in the number of endophytes recovered between locations (single factor ANOVA, *F*_9,80_ = 3.10, *p* < 0.01), with a positive correlation between the number of endophytes recovered from the roots of *H. murinum* and a light sandy silt soil (Pearson’s product moment correlation, *r* = 0.35, *p* < 0.05), a low (or unmeasurable) soil moisture content (*r* = 0.32, *p* < 0.05) and high soil salinity (*r* = 0.28, *p* < 0.05).

**Table 1.** Environmental and plant variables for collection sites.

	Soil measurements				Plant measurements				
Location	pH	Moisture content (%)	Salinity*	Soil type	Height (cm)	Zadoks stage	Health	Vegetation type	No. endophytes
1	7.2 ± 0.11	28.2 ± 2.0	1.28 ± 0.06	3	63 ± 0.99	61	5	4	5
2	7.3 ± 0.09	32.2 ± 4.2	1.46 ± 0.06	2	38 ± 4.34	66	2	2	10
3	7.8 ± 0.09	9.1 ± 2.6	1.18 ± 0.04	1	38 ± 2.96	61	2	1	6
4	8.0 ± 0.07	13.4 ± 3.0	1.41 ± 0.02	1	51 ± 3.68	61	5	2	12
5	7.6 ± 0.08	0 ± 3.2	1.22 ± 0.02	1	46 ± 4.07	66	1	3	16
6	7.9 ± 0.10	4.4 ± 4.2	1.39 ± 0.06	1	29 ± 2.49	59	4	5	7
7	7.9 ± 0.09	0 ± 3.8	1.45 ± 0.04	1	44 ± 2.74	61	3	3	15
8	7.7 ± 0.08	19.5 ± 4.2	1.26 ± 0.04	3	20 ± 1.78	61	5	4	1
9	7.7 ± 0.03	0 ± 2.8	1.49 ± 0.02	1	46 ± 3.98	61	5	3	23
10	7.7 ± 0.04	0 ± 2.8	1.51 ± 0.02	1	26 ± 3.59	66	3	3	14
MEANS	7.7 ± 0.08	10.7 ± 3.28	1.37 ± 0.04		40.1 ± 4.03	62.3 ± 0.83	3.5 ± 0.48		11 ± 2.04

Note: pH, moisture content %, salinity and height are mean values ± standard error (*n* = 10). *Salinity is osmotic pressure in bars.

### Phylogenetic analysis of endophytes

**Figure 1.**
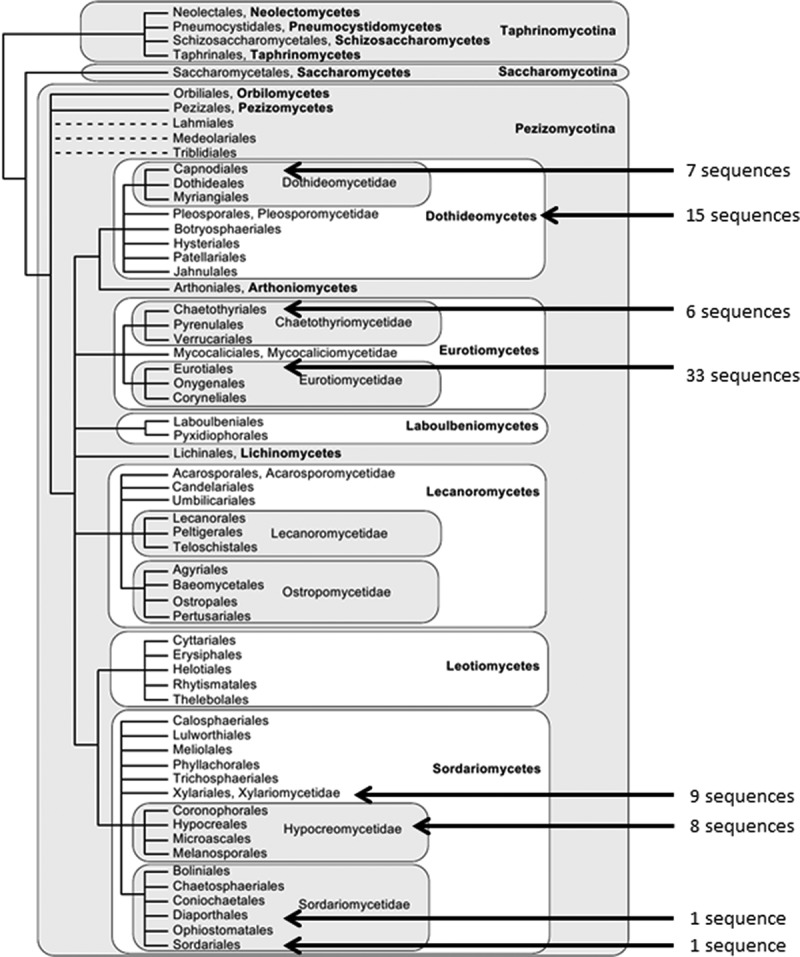
Placement of recovered endophyte isolates based on known fungal order relationships in the Pezizomycotina. Our data is overlaid on an original figure from Mycological Research, May 2007: Hibbett DS et al., “A higher-level phylogenetic classification of the Fungi”, figure 2. Copyright 2007, with permission from The British Mycological Society and Elsevier.

The 112 ITS sequences recovered from *H. murinum* roots had a mean length of 613 nucleotides, ranging from 138 to 1019. When compared to known accessions in GenBank using BLAST and our taxon designation criteria, 31 of the isolates could not be identified, with either no database match or matching an isolate with no taxonomic assignment. Eighty-one isolates could be assigned to eight fungal orders (Capnodiales, Chaetothyriales, Eurotiales, Hypocreales, Magnaporthales, Pleosporales, Sordariales and Xylariales) (Figure 1; Tables 3 and 5) with two sequences of uncertain placement (*incertae sedis*). Eighty-one sequences were categorised to the generic level, with only 34 of these assigned to the species level (Table 2). Eurotiales and Pleosporales were the most common orders, to which were respectively assigned 33 and 15 isolates. At the genus level, the most common taxon by far was *Penicillium* (27 isolates of which six were *Penicillium brevicompactum*). *Penicillium* was also the most species rich genus (excluding isolates not assigned to a species), with three separate species found. The RAT analysis reported that there were no probable recombination events detected (possible recombination events are flagged when the genetic distance in the current window is below the lower threshold parameter and either one of the next two windows is above the upper threshold parameter).

**Table 2.** Taxonomic summary of 112 fungal root endophyte isolates derived from ten Irish populations of *H. murinum*.

Fungal order	Family	Genus	Species*	Number of species
Capnodiales	Davidiellaceae	*Cladosporium*	sp.	4
	Leotiomycetidae	*Leptodontidium*	sp.	3
Chaetothyriales	Herpotrichiellaceae	*Exophiala*	*oligosperma*	4
			sp.	2
Eurotiales	Trichocomaceae	*Paecilomyces*	*marquandii*	2
			sp.	2
	Trichocomaceae	*Penicillium*	*brevicompactum*	6
			*chrysogenum*	1
			*glabrum*	2
			sp.	18
		Uncertain	sp.	2
Hypocreales	Nectriaceae	*Fusarium*	*avenaceum*	1
			*tricinctum*	3
			sp.	1
	Clavicipitaceae	*Metarhizium*	*anisopliae*	1
			sp.	2
Incertae sedis	Incertae sedis	*Cyclothyrium*	sp.	1
	Pleosporaceae	*Epicoccum*	*nigrum*	1
Magnaporthales	Magnaporthaceae	*Gaeumannomyces*	sp.	1
Pleosporales	Pleosporaceae	*Alternaria*	*tenuissima*	1
	Leptosphaeriaceae	*Coniothyrium*	sp.	1
	Montagnulaceae	*Dendrothyrium*	sp.	1
	Phaeosphaeriaceae	*Ophiosphaerella*	sp.	2
	Incertae sedis	*Phoma*	sp.	1
	Incerae sedis	*Pyrenochaeta*	*unguis-hominis*	3
			sp.	3
	Phaeosphaeriaceae	*Vrystaatia*	sp.	1
		Uncertain	sp.	2
Sordariales	Chaetomiaceae	*Chaetomium*	sp.	1
Xylariales	Incertae sedis	*Microdochium*	*bolleyi*	3
			sp.	6
Uncultured			sp.	8
Uncultured – No Match			sp.	23

Note: *Listed as species (sp.) when a sequence could be assigned with confidence to a genus but not to a species within that genus.

**Table 3.** Phylogenetic groupings of taxa in fungal orders of recovered *H. murinum* endophytes.

Fungal order	No. of species assigned sequences	No. clustering with GenBank sequence clades	No. clustering in separate clades	% clustering separately
Capnodiales	7	4	3	43
Chaetothyriales	6	4	2	33
Eurotiales	33	25	8	24
Hypocreales	8	1	7	67
Pleosporales	11	2	9	82
Xylariales	9	3	6	67
TOTALS	74	39	35	
MEANS	12	7	6	53

Note: Table is based on phylogenetic analyses of separate fungal orders (Supplementary Figures S2–S7).

For the six reconstructed phylogenies for identified genera in each fungal order (Supplementary Figures S2–S7), a mean 53% of recovered sequences did not cluster in the same clade with the selected GenBank accessions (Table 3). In particular, over half of the recovered sequences in the Hypocreales (Figure S5), Pleosporales (Figure S6) and Xylariales (Figure S7) phylogenies clustered as separate clades outside of the main generic clade. In the Hypocreales (Figure S5) all three of the recovered *Metarhizium* sequences and four of the recovered *Fusarium* sequences clustered in separate clades. In the Pleosporales (Figure S6), a clade of five recovered *Exophiala* sequences was well supported (95% bootstrap) and one recovered sequence assigned to the newly described *Vrystaatia* sp. (Quaedvlieg et al. 2013) was sister to all other clades. In the Xylariales, a clade of six recovered *Microdochium* sequences was 100% supported.

The mean distance analyses revealed large differences between the recovered sequences and the selected GenBank accessions (Table 4), with an overall difference between these two groups of 0.371 base substitutions per site. Four of the orders (Capnodiales, Chaetothyriales, Pleosporales and Xylariales) returned distances of over 0.5 base substitutions per site. The mean diversity analyses reflected this same pattern. All 112 recovered ITS sequences were deposited in GenBank and assigned accession numbers (Table 5).

**Table 4.** Mean genetic distances and diversity for endophyte ITS sequences recovered from *H. murinum* and selected GenBank ITS accessions, treated as two groups.

	Mean distance				Mean diversity			
Fungal order	Overall	Within recovered	Within GenBank	Between group	Overall	Within group	Between group	Coefficient of differentiation
Capnodiales	0.281	0.633	0.191	0.403	0.281	0.412	−0.131	−0.464
Chaetothyriales	0.273	0.606	0.159	0.468	0.273	0.382	−0.109	−0.401
Eurotiales	0.153	0.272	0.068	0.190	0.153	0.170	−0.017	−0.114
Hypocreales	0.147	0.158	0.144	0.149	0.147	0.151	−0.004	−0.029
Pleosporales	0.319	0.589	0.231	0.455	0.319	0.410	−0.091	−0.287
Xylariales	0.334	0.524	0.085	0.558	0.334	0.305	0.029	0.088
MEANS	0.251	0.464	0.146	0.371	0.251	0.305	0.064 (abs)	0.231 (abs)

Note: Figures are number of base substitutions per site, using the maximum composite likelihood model. (abs) indicates an absolute mean.

**Table 5.** List of fungal root endophyte strains isolated from wall barley (*H. murinum*), indicating assigned taxon for submission to GenBank.

Strain ID	Genus	Species	Order	GenBank accession	Closest pairwise match BLAST
040101(1)	Cladosporium		Capnodiales	KP309943	HF952649.1, 95%
040105(2)A	Uncultured	Uncultured	Uncultured	KP309980	<85%
04020703A	Paecilomyces	Marquandii	Eurotiales	KP309881	JQ013003.1, 99%
04020703B	Paecilomyces		Eurotiales	KP309882	JQ846086.1, 93%
0402073A	Paecilomyces	Marquandii	Eurotiales	KP309918	JQ013005.1, 98%
040406(4)	Penicillium		Eurotiales	KP309981	KJ775685.1, 91%
04040603A	Uncultured	Uncultured	Uncultured	KP309883	KM494487.1, 88%
040510(3)	No significant	No significant	No significant	KP309982	<85%
040605(2)CB	No significant	No significant	No significant	KP309983	<85%
04060502A	Cladosporium		Capnodiales	KP309884	KM492836.1, 99%
04060502AA	Uncultured	Uncultured	Uncultured	KP309903	<85%
04060502B	Cladosporium		Capnodiales	KP309885	KM492836.1, 98%
04060502BB	No significant	No significant	No significant	KP309904	<85%
04060502C	No significant	No significant	No significant	KP309905	<85%
040706(1)	Exophiala	Oligosperma	Chaetothyriales	KP309944	AB777520.1, 99%
040901(2)	Alternaria	Tenuissima	Pleosporales	KP309984	KF516939.1, 97%
040901(3)C	Penicillium		Eurotiales	KP309985	EF682113.1, 95%
04090103A	Penicillium		Eurotiales	KP309886	JX869555.1, 95%
04090103B	Penicillium		Eurotiales	KP309887	KM492842.1, 95%
04090205A	Pyrenochaeta	Unguis-hominis	Pleosporales	KP309888	KP132547.1, 98%
04090205B	Pyrenochaeta		Pleosporales	KP309889	KC113302.1, 96%,
040903(1)	Penicillium		Eurotiales	KP309986	KM189631.1, 92%
04090503A	Pyrenochaeta	Unguis-hominis	Pleosporales	KP309890	KP132547.1,98%
04090503B	Pyrenochaeta	Unguis-hominis	Pleosporales	KP309891	KP132548.1, 98%
04090604A	Metarhizium		Hypocreales	KP309892	EU307928.1, 93%
04090604B	Metarhizium		Hypocreales	KP309893	AY646397.1, 92%
04090604D	Metarhizium	Anisopliae	Hypocreales	KP309936	KC140227.1, 98%
040907(5)	Pyrenochaeta		Pleosporales	KP309945	JX966641.1, 95%
040909(5)	Paecilomyces		Eurotiales	KP309987	JQ013005.1, 92%
04090905B	No significant	No significant	No significant	KP309894	<85%
04100405A	Exophiala	Oligosperma	Chaetothyriales	KP309895	AB777520.1, 99%
04100405B	Exophiala	Oligosperma	Chaetothyriales	KP309896	KM492841.1, 99%
04100405E	No significant	No significant	No significant	KP309910	<85%
0410045A	Penicillium	Brevicompactum	Eurotiales	KP309937	JX869555.1, 97%
041008(5)E	Uncultured	Uncultured	Uncultured	KP309946	JN847480.1, 96%
04100805A	Penicillium	Brevicompactum	Eurotiales	KP309897	KM492842.1, 97%
04100805B	No significant	No significant	No significant	KP309898	<85%
0410085D			Eurotiales	KP309922	KJ135335.1, 89%
04101003A	Penicillium		Eurotiales	KP309899	DQ123637.1, 95%
04101003B	Penicillium		Eurotiales	KP309900	DQ249211.1, 92%
041103(3)B	No significant	No significant	No significant	KP309947	<85%
041106(1)B	No significant	No significant	No significant	KP309948	<85%
0411061A	Penicillium		Eurotiales	KP309924	DQ888735.1, 95%
0411061AA			Eurotiales	KP309925	KF018417.1, 86%
041107(2)A	Microdochium	Bolleyi	Xylariales	KP309949	KF646098.1, 99%
041108(4)A	Penicillium	Glabrum	Eurotiales	KP309988	KM396380.1, 98%
041109(1)B	No significant	No significant	No significant	KP309950	<85%
0412062A	Penicillium		Eurotiales	KP309926	GU134895.1, 88%
0412062B	Penicillium		Eurotiales	KP309927	JN986785.1, 86%
0412065A	Penicillium		Eurotiales	KP309939	GU134895.1, 94%
0412065B	Penicillium	Chrysogenum	Eurotiales	KP309940	KF572142.1, 99%
0412074A	Fusarium		Hypocreales	KP309928	JX406512.1, 95%
0412074B	Fusarium	Avenaceum	Hypocreales	KP309929	DQ093676.1, 99%
0412083A	Fusarium	Tricinctum	Hypocreales	KP309941	JX406512.1, 99%
0412083B	Fusarium	Tricinctum	Hypocreales	KP309942	KJ598871.1, 99%
4030202	No significant	No significant	No significant	KP309901	<85%
404063	Uncultured	Uncultured	Uncultured	KP309919	HQ022030.1, 87%
4060605			Pleosporales	KP309906	HM116751.1, 85%
4090205			Pleosporales	KP309907	KF800200.1, 84%
4090503	No significant	No significant	No significant	KP309908	<85%
4090604	Vrystaatia		Pleosporales	KP309909	KF251278.1, 96%
409094	Pyrenochaeta		Pleosporales	KP309920	KP132548.1, 94%
4040905A	Penicillium		Eurotiales	KM492840	AY484931.1, 93%
410021	Cyclothyrium		Incertae sedis	KP309921	FJ025227.1, 97%
4100801	Dendrothyrium	Variisporum	Pleosporales	KP309911	JX496053.1, 96%
410103	Penicillium	Brevicompactum	Eurotiales	KP309923	KJ775605.1, 98%
412063	Fusarium	Tricinctum	Hypocreales	KP309938	JX406511.1, 98%
GgtA	Exophiala	Oligosperma	Chaetothyriales	KP309912	KJ652929.1, 98%
IA51015A	No significant	No significant	No significant	KP309930	<85%
IA51015B	No significant	No significant	No significant	KP309931	<85%
IA54031	Penicillium	Brevicompactum	Eurotiales	KP309932	JX869555.1, 98%
IA54042	Penicillium	Brevicompactum	Eurotiales	KP309933	JX156371.1, 99%
IA72	Penicillium sp.		Eurotiales	KP309913	AJ877044.1, 97%
IA73	Coniothyrium		Pleosporales	KP309914	AM901685.1, 99%
IA76			Pleosporales	KP309915	HM116751.1, 85%
IA93	Penicillium		Eurotiales	KP309916	GU441578.1, 90%,
Isolate 1	Microdochium		Xylariales	KP309952	KC989068.1, 95%
Isolate 10A	Leptodontidium		Capnodiales	KP309963	JX077084.1, 91%
Isolate 10B	Leptodontidium		Capnodiales	KP309976	KJ188690.1, 96%
Isolate 11A			Eurotiales	KP309964	KM189631.1, 90%
Isolate 11B	Penicillium	Glabrum	Eurotiales	KP309977	KM189631.1, 99%
Isolate 12A	Chaetomium		Sordariales	KP309965	KJ186956.1, 95%
Isolate 12B	Cladosporium		Capnodiales	KP309978	AM262400.1, 98%
Isolate 13A	No significant	No significant	No significant	KP309966	<85%
Isolate 13B	Leptodontidium		Capnodiales	KP309979	KJ188690.1, 92%
Isolate 14A	No significant	No significant	No significant	KP309967	<85%
Isolate 1A	No significant	No significant	No significant	KP309951	<85%
Isolate 2	Microdochium		Xylariales	KP309954	AB517933.1, 97%
Isolate 2A	Gaeumannomyces		Magnaporthales	KP309953	KM484834.1, 93%
Isolate 3A	Epicoccum		Incertae sedis	KP309955	AJ279452.1, 91%
Isolate 3B	Epicoccum	Nigrum	Incertae sedis	KP309970	GU934519.1, 99%
Isolate 4A	Uncultured	Uncultured	Uncultured	KP309956	JX321359.1, 87%
Isolate 4B	Microdochium	Bolleyi	Xylariales	KP309968	GU934540.1, 98%
Isolate 5A	Ophiosphaerella		Pleosporales	KP309957	AJ246157.1, 96%
Isolate 5B	Microdochium	Bolleyi	Xylariales	KP309969	JQ658340.1, 98%
Isolate 5BB	Ophiosphaerella		Pleosporales	KP309971	KC694154.1, 98%
Isolate 6A	No significant	No significant	No significant	KP309958	<85%
Isolate 6B	No significant	No significant	No significant	KP309972	<85%
Isolate 7A	Microdochium		Xylariales	KP309959	JQ658340.1, 94%
Isolate 7B	No significant	No significant	No significant	KP309973	<85%
Isolate 8	Microdochium	Bolleyi	Xylariales	KP309961	KC989068.1, 99%
Isolate 8A	Microdochium		Xylariales	KP309960	JX280599.1, 93%
Isolate 8B	Microdochium		Xylariales	KP309974	JX368718.1, 95%
Isolate 9A	No significant	No significant	No significant	KP309962	HM997116.1, 83%
Isolate 9B	No significant	No significant	No significant	KP309975	<85%
Loc09A	Penicillium		Eurotiales	KP309902	EU587326.1, 84%
MPJ012	Exophiala		Chaetothyriales	KP309934	KF928424.1, 89%
MPJ012A	Exophiala		Chaetothyriales	KP309935	KP132110.1, 89%
PPUNK01	No significant	No significant	No significant	KP309917	<85%

Only one significant correlation was found between any taxon level and environmental characteristics of the sample source sites. We found a strong positive correlation (*r* = 0.69, *p* < 0.01) between high soil salinity and the number of *Penicillium* isolates recovered.

## Discussion

We recovered a total of 112 ascomycete fungal isolates from *H. murinum* which can be divided into 8 orders, 12 families and 18 genera. The number of diverse and novel endophytes recovered from this wild relative of cultivated barley may only be the tip of the iceberg. They are spread across Ascomycota but were not detected in Saccharomycotina, Taphrinomycotina, the most outlying lineages of Pezizomycotina (Figure 1), or outside of Ascomycota in for example its Dikarya sister group, the Basidiomycota. Some other authors have indicated that, to date, only a small fraction of all endophytes have been detected (Anderson and Cairney 2004; Porras-alfaro and Bayman 2011; Weiss et al. 2011). The methods to rapidly detect them have only recently emerged. Furthermore, the problem is magnified because many endophytes are unculturable outside of their host (Allen et al. 2003). Even those that do emerge from roots onto artificial media may not be easily cultured or may not sporulate readily. For this study, we isolated only those endophytes that can be grown and multiplied in standard artificial media and that may have potential as inoculants for performance improvement in cultivated barley. A high proportion of novel fungi was revealed in our study, with 28% not assigned to a fungal order (Table 2), suggesting a high degree of diversity still to be discovered in this class of microorganisms. The true diversity of endophytes in our root samples may be under-estimated as we only used a single culture medium and cultural condition for recovery and isolation. Similar studies have tested either a different medium or a range of media under different cultural conditions. Verma et al. (2011) found that some of the endophytic isolates were only recovered on certain media, while other researchers have chosen a different culture medium than ours (de Souza Leite et al. 2013; Fernandes et al. 2015). Further experimental work with a range of culture media and cultural conditions is necessary to determine the full diversity of endophytes in our selected host. As considerable benefits to barley induced by a small number of these particular endophytes have already been shown (Murphy et al. 2015a, 2015b), the agricultural and horticultural potential for other undescribed and untested endophytes may be vast.

### Endophyte ecology

The sites where the populations of the endophyte host, *H. murinum*, occurred may be important determinants of the particular composition of the recruited endophytes that we have recovered for our study. As these sites were characterised by small populations (<50 individuals), a relatively open and dry environment, shallow sandy soil and a lack of vegetative competition, then the endophytes may be associated with stress tolerance in the host (Rodriguez et al. 2008). Even though the measured site and plant parameters indicated that the plants were growing under at least abiotic stress, most of the plants were in reasonably good health (only three populations were classed as being in poor health, and these were not on the most saline soils) and all had flowered.

### Phylogenetic relationships

When assigning taxon identity from a DNA sequence it is important to understand that similarity-based taxonomic assignment is impeded by differences between the unassigned read and reference database, forcing a rank-specific classification to the closest reference lineage (Porter and Beiko 2013). While efforts to develop and improve search algorithms for reference database such as GenBank have addressed many of these issues (Altschul et al. 1997; Porter and Beiko 2013) the NCBI megaBLAST suite provides a reliable and robust set of tools to enable accurate taxon assignment. We used conservative criteria when assigning taxon identities using BLAST and assessed all of the pairwise similarity matches for each search before assigning an identity, but even so we could still not be sure of the accuracy of some GenBank accession taxon names. The ITS sequence identities recovered from GenBank in our study that did not cluster within the clades containing their taxonomic counterparts may not have been correctly identified when they were originally deposited. Many sequences deposited in GenBank are associated with erroneous taxon names (Nilsson et al. 2006). While the quality and reliability of DNA sequences in public databases may be improving, some GenBank sequences may not be reliable, with as much as 86% of available fungal sequences not from the named organism (Ko Ko et al. 2011).

Despite our efforts to assign organism identities to the endophyte sequences, the main finding from the BLAST searches was the strikingly low mean pairwise similarity match, with only 34 sequences (30% of the total) assigned to the species level. The high number of sequence searches which returned no significant match indicates that *H. murinum* harbours a large number of novel and undescribed culturable endophytes. The reconstructed phylogenies and the distance and diversity analyses for each of the analysed fungal orders support this conclusion, with many of the recovered sequences clustering as separate clades outside of the main generic group. Chen et al. (2015) suggest that in the Eurotiomycetes the evolution of fungal endophytism might be concentrated in three orders instead of occurring widely in every lineage of the class, and our results support this view. We see the same assignment of the recovered endophytes into relatively few orders within each of the other classes (Figure 1). Chen et al. (2015) also suggest that undescribed endophytic fungi in the Eurotiomycetes may constitute a new order closely related to Chaetothyriales and Eurotiales. This may explain part of the phylogenetic out-placement of recovered Eurotiomycetous endophytes in our study, and could extend to the other classes. Examination of the morphological characters of mycelia and/or spores of the unidentified fungi revealed no obvious affinities that could confidently assign identity, providing the opportunity of describing possible new species in the future.

Of those sequences that could be confidently assigned to the genus level, the most common taxon by far was *Penicillium*, and the number of *Penicillium* isolates recovered was significantly correlated with a high soil salinity. The endophytes identified to species level here are mostly common soil fungi and represent a broad mix of known pathogenic and beneficial fungi. While *Penicillium* is one of the most commonly occurring fungi in the rhizosphere, the relatively high soil salinity at all of the sampling sites may account for the predominance of this salt-tolerant genus in our study (El-Mougith 1993). One sequence assigned to the newly described *Vrystaat* sp. (Pleosporales, Phaeosphaeriaceae) (Quaedvlieg et al. 2013) was sister to all other clades. This *Septoria*-like fungus was originally isolated and so far only described from the decaying leaf-tips of an *Aloe* sp. in South Africa, so it is perhaps surprising to find it here.

Similar endophyte surveys have reported varied and diverse assemblages of fungal taxa, depending on the host and environment. A recent study which sampled the endophyte diversity in the above-ground tissues of cotton from several populations found a very diverse group, but with relatively few *Penicillium* (Ek-Ramos et al. 2013). Fungal diversity was also high in a review of the endophytes in grass species, with some assemblage similarity to ours (Sánchez Márquez et al. 2012). The only study which specifically focused on characterising fungal root endophytes from a single species (tomato, *Solanum lycopersicon*) also revealed the presence of many putative new species (Andrade-Linares et al. 2011). The presence of many other unculturable endophytes in many plants is likely, with even relatively new single orders of endophyte found to be ubiquitous (Weiss et al. 2011). Saunders et al. (2010) found that different soils could act as different habitat filters for endophytes, and we have also found this to be the case.

### Potential of endophytes in agriculture

Cytogenetic studies of meiotic chromosome behaviour in interspecific hybrids led to the definition of four different genomes within *Hordeum* (Blattner 2009). Though *H. murinum* and the crop species *H. vulgare* (and derived cultivars) belong to different genome groups, the generic similarity may indicate a beneficial potential for endophytes isolated from *H. murinum*. Hybrids have been produced between *H. murinum* and *H. vulgare* using tissue culture techniques (Jorgensen et al. 1986) but the progeny are sterile. Murphy et al. 2015a, 2015b) have already demonstrated the benefits to barley induced by at least 10 of the endophytes studied here, so many of the others may be equally competent. They are easily cultured, are proven survivors/competitors in stressed field conditions and locally adapted so may be particularly useful in co-local barley crops. Further studies with wild crop relatives of other cereal crops may reveal similar benefits for their cultivated relatives. As barley is the fourth most important global cereal crop, grown annually on 48 million hectares (CGIAR 2012), the potential economic and environmental benefits derived from the use of the fascinating, largely undescribed and diverse group of genus-associated endophytes as crop inoculants may be great.

Our results suggest that there is potential for investigating the use of some of these endophytes as barley crop inoculants on high pH soils. Many *Hordeum* species are tolerant of relatively high soil salinity, and *H. murinum* is no exception (Garthwaite et al. 2005). The populations of *H. murinum* in our study were all growing in alkaline, saline soils, so endophytes derived from *H. murinum* growing in alkaline soils may confer greater salt tolerance in *H. vulgare* cultivars.

## Disclosure statement

No potential conflict of interest was reported by the authors.

## Supplemental data

Supplemental data for this article can be accessed at http://dx.doi.10.1080/21501203.2015.1070213.

Supplementary_material.zip
