# Supplementary material for: Profundae diversitas: the uncharted genetic diversity in a newly studied group of fungal root endophytes
Source: Mycology. 2015 Jul 24;6(3-4):139–50. doi: 10.1080/21501203.2015.1070213 (PMC6106079; doi:10.1080/21501203.2015.1070213)
Supplement: Supplementary_material.zip [file TMYC_A_1070213_SM8098.zip › Supplementary material/Figure captions.docx]

# Figure captions

**Figure S2.** Maximum Likelihood phylogenetic reconstruction of recovered Capnodiales nrITS sequences and selected GenBank accessions, using the Kimura 2- parameter model. Recovered sequences from the current study are in italics and prefixed TCD14E. Figures at branches are bootstrap values.

**Figure S3.** Maximum Likelihood phylogenetic reconstruction of recovered Chaetothryales nrITS sequences and selected GenBank accessions, using the Kimura 2- parameter model. Recovered sequences from the current study are in italics and prefixed TCD14E. Figures at branches are bootstrap values.

**Figure S4.** Maximum Likelihood phylogenetic reconstruction of recovered Eurotiales nrITS sequences and selected GenBank accessions, using the Kimura 2- parameter model. Recovered sequences from the current study are in italics and prefixed TCD14E. Figures at branches are bootstrap values, showing only those bootstrap values over 0.5.

**Figure S5.** Maximum Likelihood phylogenetic reconstruction of recovered Hypocreales nrITS sequences and selected GenBank accessions, using the Kimura 2- parameter model. Recovered sequences from the current study are in italics and prefixed TCD14E. Figures at branches are bootstrap values.

**Figure S6.** Maximum Likelihood phylogenetic reconstruction of recovered Pleosporales nrITS sequences and selected GenBank accessions, using the Kimura 2- parameter model. Recovered sequences from the current study are in italics and prefixed TCD14E. Figures at branches are bootstrap values.

**Figure S7.** Maximum Likelihood phylogenetic reconstruction of recovered Xylariales nrITS sequences and selected GenBank accessions, using the Kimura 2- parameter model. Recovered sequences from the current study are in italics and prefixed TCD14E. Figures at branches are bootstrap values.
