# Supplementary figures and images for: Profundae diversitas: the uncharted genetic diversity in a newly studied group of fungal root endophytes
Source: Mycology. 2015 Jul 24;6(3-4):139–50. doi: 10.1080/21501203.2015.1070213 (PMC6106079; doi:10.1080/21501203.2015.1070213)

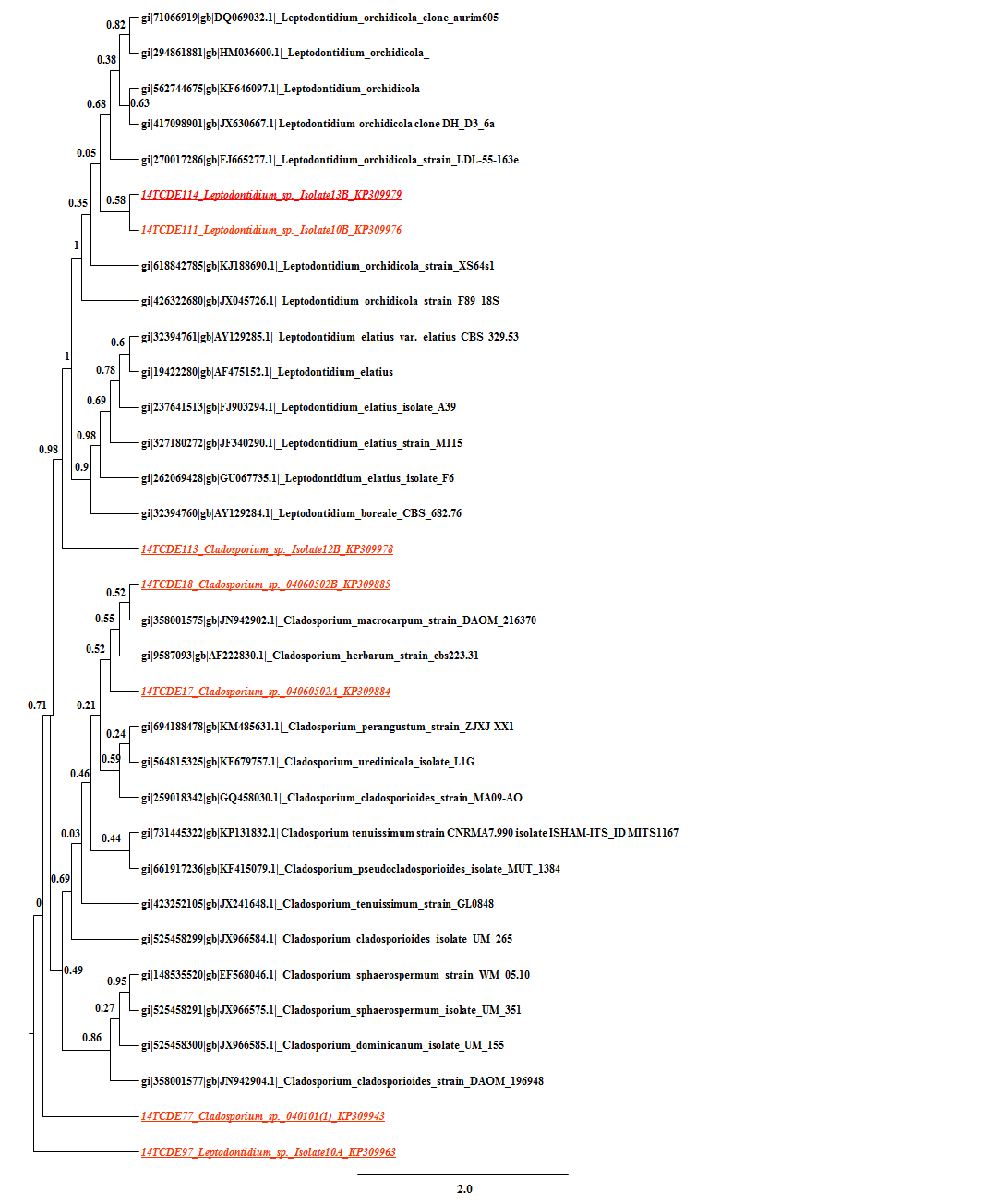

Supplement: Supplementary_material.zip [file TMYC_A_1070213_SM8098.zip › Supplementary material/Figure_S2.tif]

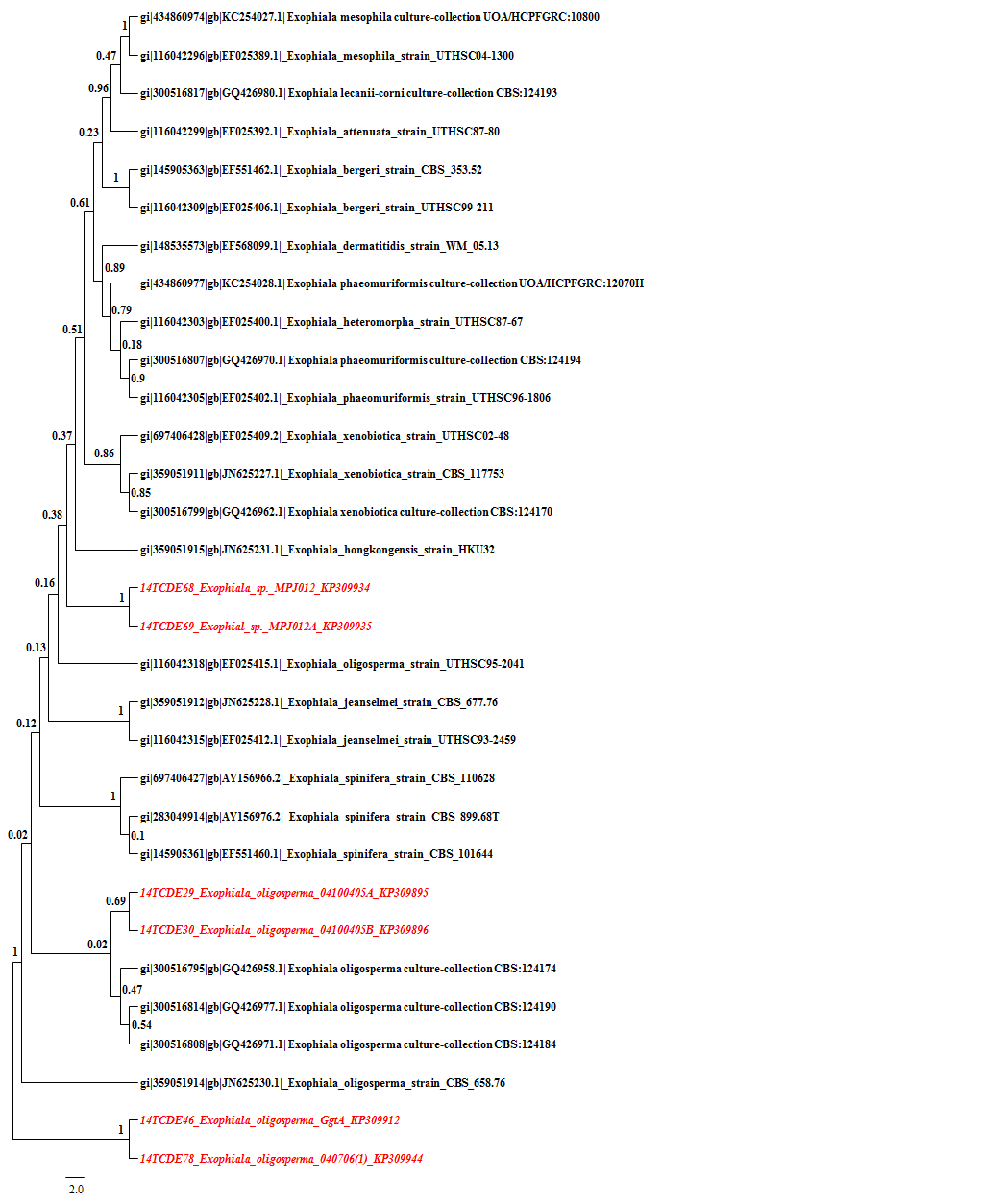

Supplement: Supplementary_material.zip [file TMYC_A_1070213_SM8098.zip › Supplementary material/Figure_S3.tif]

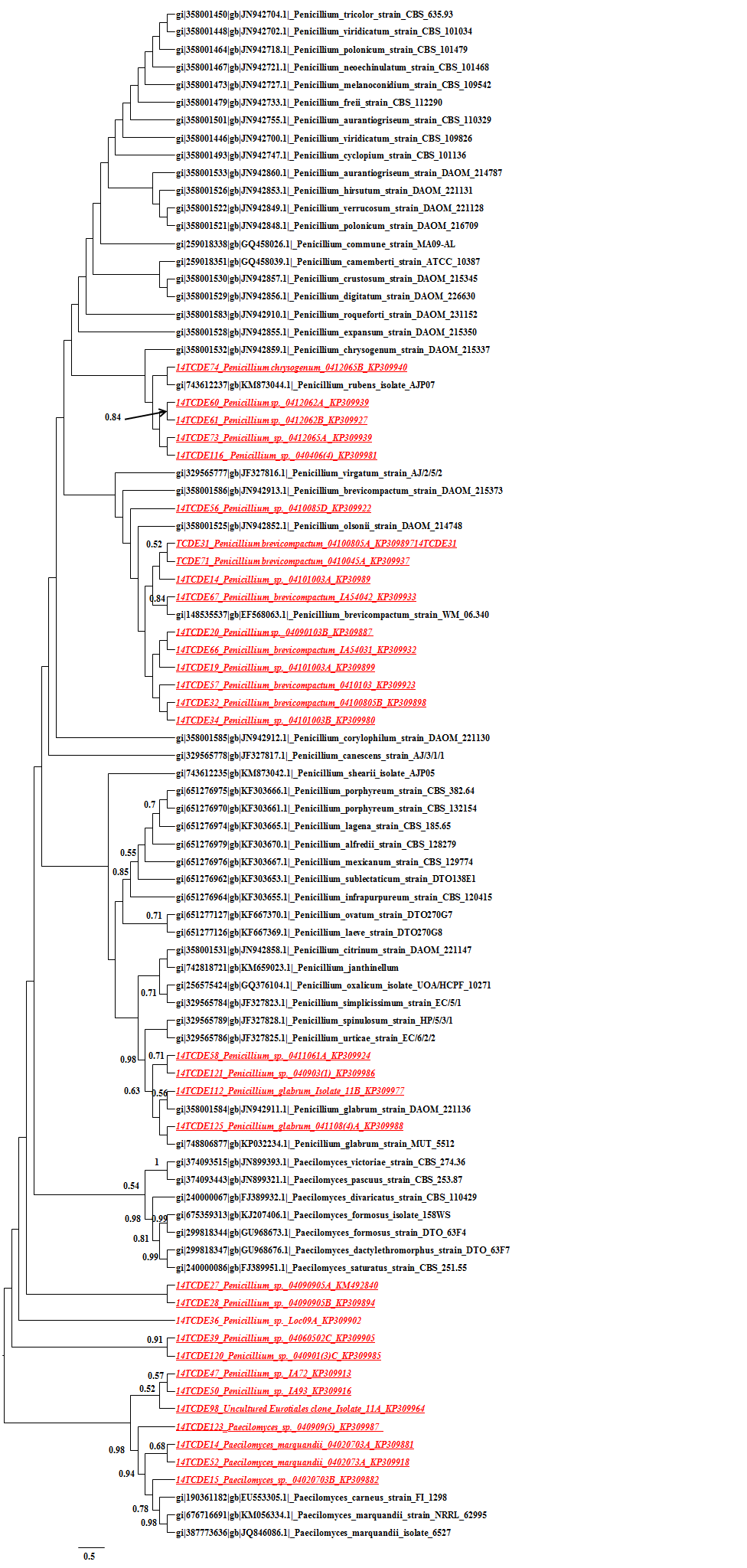

Supplement: Supplementary_material.zip [file TMYC_A_1070213_SM8098.zip › Supplementary material/Figure_S4.tif]

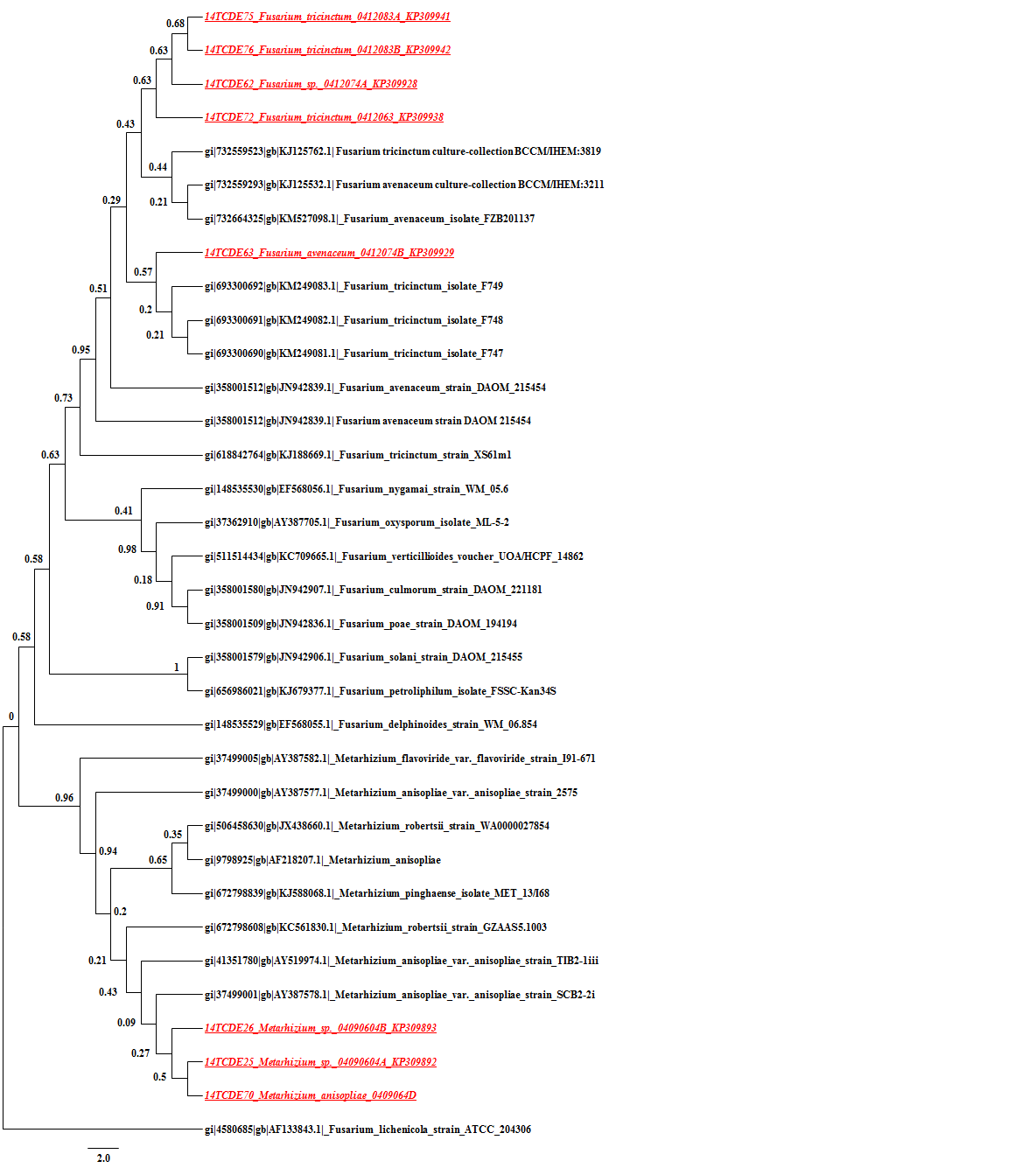

Supplement: Supplementary_material.zip [file TMYC_A_1070213_SM8098.zip › Supplementary material/Figure_S5.tif]

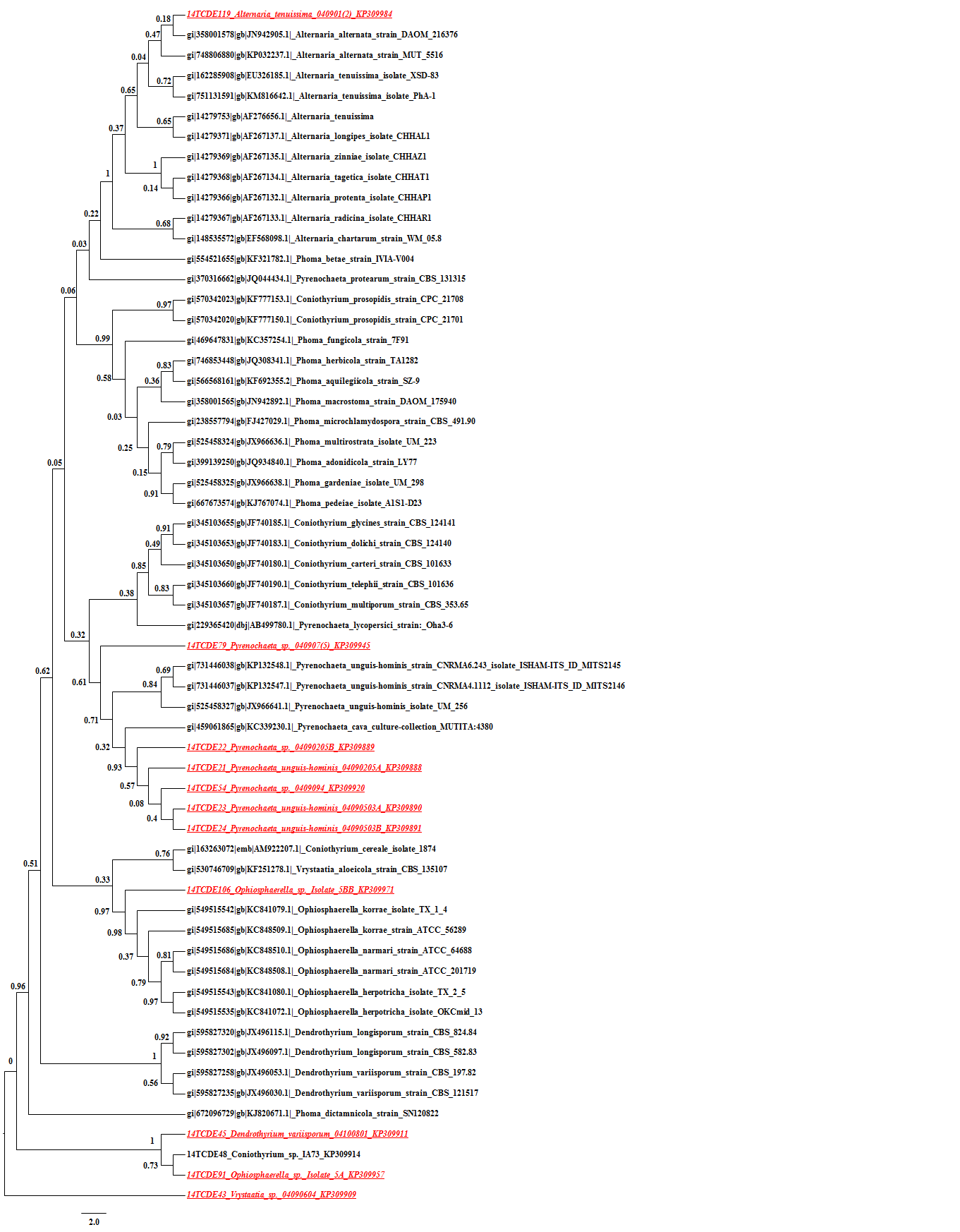

Supplement: Supplementary_material.zip [file TMYC_A_1070213_SM8098.zip › Supplementary material/Figure_S6.tif]

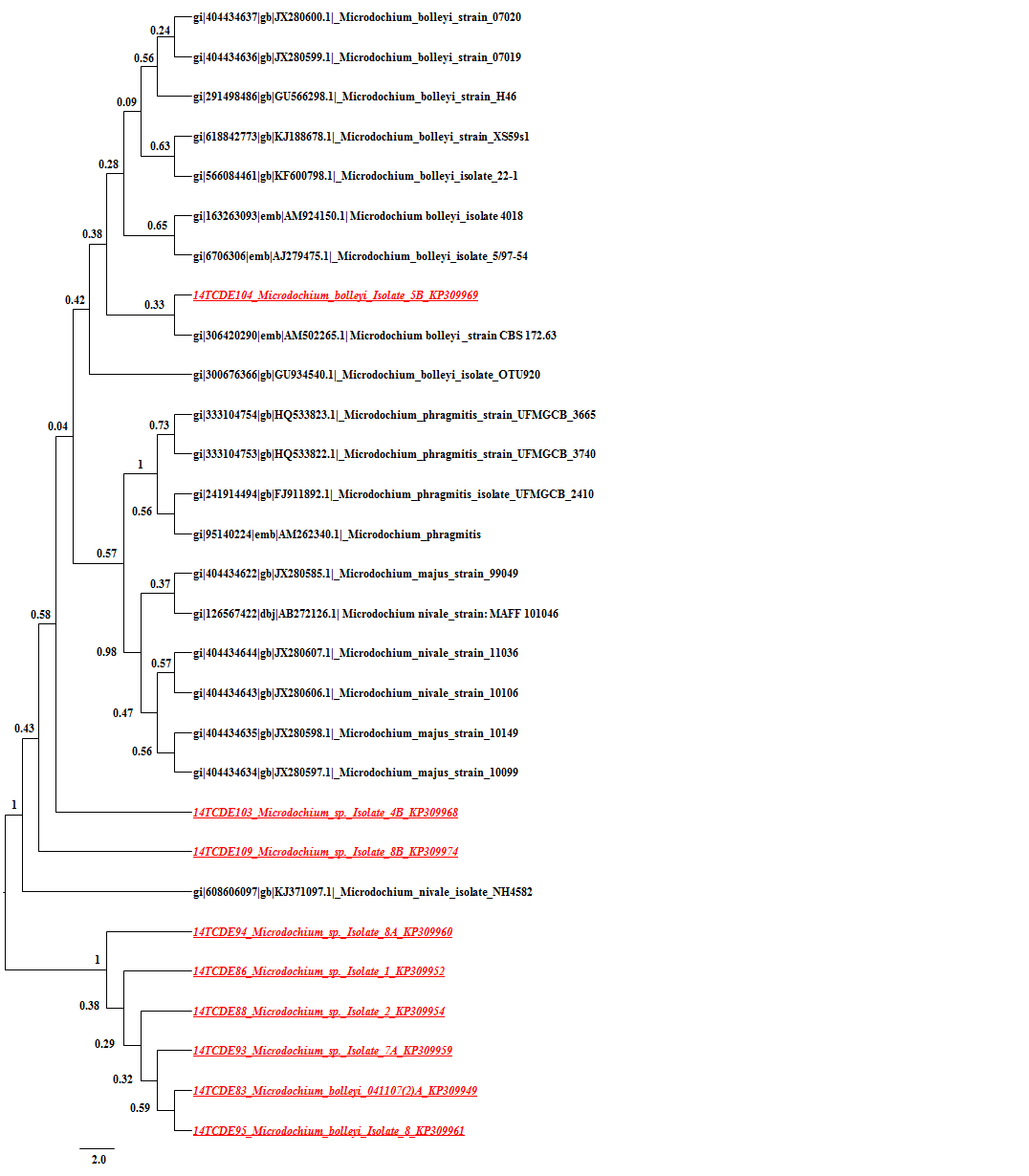

Supplement: Supplementary_material.zip [file TMYC_A_1070213_SM8098.zip › Supplementary material/Figure_S7.tif]
